# UDP-sulfoquinovose formation by *Sulfolobus acidocaldarius*

**DOI:** 10.1007/s00792-015-0730-9

**Published:** 2015-01-21

**Authors:** Behnam Zolghadr, Bernhard Gasselhuber, Markus Windwarder, Martin Pabst, Daniel Kracher, Martina Kerndl, Sonja Zayni, Andreas Hofinger-Horvath, Roland Ludwig, Dietmar Haltrich, Chris Oostenbrink, Christian Obinger, Paul Kosma, Paul Messner, Christina Schäffer

**Affiliations:** 1 Department of NanoBiotechnology, NanoGlycobiology Unit, Universität für Bodenkultur Wien, Muthgasse 11, 1190 Vienna, Austria; 2 Department of Chemistry, Universität für Bodenkultur Wien, Muthgasse 18, 1190 Vienna, Austria; 3 Department of Food Science and Technology, Universität für Bodenkultur Wien, Muthgasse 11, 1190 Vienna, Austria; 4 Institute for Molecular Modeling and Simulation, Universität für Bodenkultur Wien, Muthgasse 18, 1190 Vienna, Austria; 5 Present Address: Department of Chemistry and Applied Biosciences, ETH Zürich, HCI D 330 Vladimir-Prelog-Weg 1-5/10, 8093 Zurich, Switzerland

**Keywords:** UDP-sulfoquinovose synthase Agl3, *Sulfolobus acidocaldarius*, Enzyme mechanism, Site-directed mutagenesis, Sulfite

## Abstract

The UDP-sulfoquinovose synthase Agl3 from *Sulfolobus acidocaldarius* converts UDP-d-glucose and sulfite to UDP-sulfoquinovose, the activated form of sulfoquinovose required for its incorporation into glycoconjugates. Based on the amino acid sequence, Agl3 belongs to the short-chain dehydrogenase/reductase enzyme superfamily, together with SQD1 from *Arabidopsis thaliana*, the only UDP-sulfoquinovose synthase with known crystal structure. By comparison of sequence and structure of Agl3 and SQD1, putative catalytic amino acids of Agl3 were selected for mutational analysis. The obtained data suggest for Agl3 a modified dehydratase reaction mechanism. We propose that in vitro biosynthesis of UDP-sulfoquinovose occurs through an NAD^+^-dependent oxidation/dehydration/enolization/sulfite addition process. In the absence of a sulfur donor, UDP-d-glucose is converted via UDP-4-keto-d-glucose to UDP-d-glucose-5,6-*ene*, the structure of which was determined by ^1^H and ^13^C-NMR spectroscopy. During the redox reaction the cofactor remains tightly bound to Agl3 and participates in the reaction in a concentration-dependent manner. For the first time, the rapid initial electron transfer between UDP-d-glucose and NAD^+^ could be monitored in a UDP-sulfoquinovose synthase. Deuterium labeling confirmed that dehydration of UDP-d-glucose occurs only from the enol form of UDP-4-keto-glucose. The obtained functional data are compared with those from other UDP-sulfoquinovose synthases. A divergent evolution of Agl3 from *S.* *acidocaldarius* is suggested.

**Electronic supplementary material:**

The online version of this article (doi:10.1007/s00792-015-0730-9) contains supplementary material, which is available to authorized users.

## Introduction

UDP-sulfoquinovose is the nucleotide-activated form of sulfoquinovose (6-deoxy-6-*C*-sulfo-d-glucopyranose, Qui6S) and is required for the incorporation of sulfoquinovose into glycoconjugates. Among those is, for instance, the sulfolipid sulfoquinovosyl diacylglycerol which is found in the chloroplast membrane of plants and in cyanobacteria (Benning et al. 1993; Benning 1998; Riekhof et al. 2003; Sato et al. 2003; Shimojima 2011; Denger et al. 2014), with cyanobacterial strains of the genus *Spirulina* having recently gained interest because of the anti-HIV properties of some of their sulfoquinovose-containing sulfolipids (Kwei et al. 2011). In the hyperthermophilic archaeon *Sulfolobus acidocaldarius* sulfoquinovose is either a component of the glycosylated, membrane-associated cytochrome *b* complex (Zähringer et al. 2000), the major surface (S-) layer protein SlaA (Peyfoon et al. 2010), or the subunit of the archaellum filament FlaB (Meyer et al. 2013). Further reports on sulfoquinovose in archaea concern a so far uncharacterized oligosaccharide modifying the S-layer protein of *Haloferax volcanii* (Eichler 2013; Parente et al. 2014) and an operon encoding a sulfoquinovose synthase (SqdB) in the haloarchaeon *Haloquadratum walsbyi* (Bolhuis et al. 2006).

Work on the sulfolipid biosynthesis in *Arabidopsis thaliana* identified SQD1 as the biosynthesis enzyme for UDP-sulfoquinovose (Benning et al. 1993; Benning 1998), with SQD1 currently being the best-investigated UDP-sulfoquinovose-synthase. It shows high sequence similarity to sulfolipid biosynthesis enzymes of different organisms and to sugar nucleotide modifying enzymes such as UDP-glucose epimerase GalE (Thoden et al. 1996a; Liu et al. 1997) and dTDP-glucose-4,6-dehydratase (Gross et al. 2000; Allard et al. 2002). In a three-dimensional model of SQD1, which is based on the 1.8-Å crystallographic structure of UDP-glucose 4-epimerase (Liu et al. 1997) as a template, an NAD^+^ binding site and active site interactions were predicted (Essigmann et al. 1999). The proposed reaction mechanism of SQD1 was confirmed after its crystallization at 1.6-Å resolution in a complex with NAD^+^ and the putative substrate UDP-d-glucose (Mulichak et al. 1999). The SQD1 protein has a bi-domain structure with a Rossmann fold for NAD^+^ binding, revealing high structural similarity with the GalE enzyme (Thoden et al. 1996b). It is a member of the SDR family of enzymes (Kavanagh et al. 2008), with its structure showing conservation of the catalytic SDR amino acid residues. The Rossmann-fold fingerprint sequence at the pyrophosphate binding site of SDR enzymes is replaced by a G-XX-G-XX-G sequence in SQD1 (Mulichak et al. 1999), while the characteristic Y-XXX-K motif and a Ser/Thr residue are located at the active site of SQD1, to form the catalytic triad of SDR enzymes (Kavanagh et al. 2008) with Thr145, Tyr182, and Lys186 (Mulichak et al. 1999).

The proposed mechanism for SQD1 catalysis suggests that, in the absence of a sulfur donor, the reaction continues to the UDP-4-keto-glucose-5,6-*ene* product. At the active site of the enzyme, UDP-d-glucose and NAD^+^ are bound, with the latter in the oxidized state. In a subsequent step, a sulfur donor would transfer sulfite to UDP-4-keto-glucose-5,6-*ene* by a nucleophilic addition across the double bond, followed by reduction of the 4-keto group and regeneration of NAD^+^ (Mulichak et al. 1999).

Characterization of SQD1 from *A.* *thaliana* (Sanda et al. 2001) showed that the highly purified enzyme exists as a complex with ferredoxin-dependent glutamate synthase (Shimojima et al. 2005), and the crystal structure of SQD1 showed that the NAD^+^ cofactor is tightly bound to the N-terminal domain of the enzyme (Mulichak et al. 1999). The main bottleneck for fully elucidating the mechanism of SQD1 was its low in vitro activity. Recombinant SQD1 expressed in *Escherichia coli* showed low in vitro activity as well (Sanda et al. 2001). Thus, the in vivo-mechanism of the sulfite transfer to C-6 of UDP-d-glucose by UDP-sulfoquinovose synthases is still unknown.

To elucidate the biosynthesis of UDP-sulfoquinovose in *S.* *acidocaldarius*, its genome was scanned for the presence of homologues of the bacterial *sqdB* or eukaryal *sqd1* genes known to encode UDP-sulfoquinovose synthases (Meyer et al. 2011). The scan revealed Saci0423 with ~40 % sequence identity to SQD1 of *A. thaliana*. As shown for SQD1, Agl3 is a member of the SDR superfamily of enzymes (Field and Naismith 2003; Kavanagh et al. 2008). In a recent study, the *agl3* (*saci0423*) gene was confirmed to code for the UDP-sulfoquinovose synthase involved in the biosynthesis of the *S. acidocaldarius* S-layer *N*-glycan. Targeted deletion of *agl3* impaired UDP-sulfoquinovose synthesis and resulted in a mutant lacking sulfoquinovose in its S-layer glycan (Meyer et al. 2011). In addition, the lack of *agl3* resulted in a reduced molecular mass of FlaB, indicating that FlaB is also modified with the *N*-glycan containing sulfoquinovose (Meyer et al. 2013). In close vicinity of *agl3*, several genes are localized which are predicted to code for carbohydrate-active enzymes linked to the UDP-sulfoquinovose metabolism. These include *agl4* (*saci0424*), annotated as a glucokinase, predicted to provide glucose 1-phosphate as a substrate for the NDP-glucose pyrophosphorylase Agl2, encoded by *agl2* (*saci0422*), which would generate UDP-glucose, serving as the immediate substrate for Agl3. Eventually, *agl1* (*saci0421*), coding for a membrane-bound glycosyltransferase, would be involved in the transfer of sulfoquinovose from UDP-sulfoquinovose to the *N*-acetylglucosamine residue of the *N*-glycan (Meyer et al. 2011).

In the present study, we investigate the in vitro activity of Agl3 and propose that different reaction products can be formed dependent on the type and the amount of substrate. A complex reaction cycle is followed, including oxidation, dehydration, enolization, and reprotonation of UDP-d-glucose. Point mutation studies have been performed by targeting amino acids known to be crucial for enzymatic activity of enzymes from the SDR superfamily (Field and Naismith 2003; Kavanagh et al. 2008). This study provides new insights into the reaction mechanism of Agl3 of *S. acidocaldarius* and additionally unveils possible evolutionary differences between the planta enzyme SQD1 and the prokaryotic UDP-sulfoquinovose synthase.

## Results and discussion

### Phylogenetic and functional analysis of Agl3

**Fig. 1:**
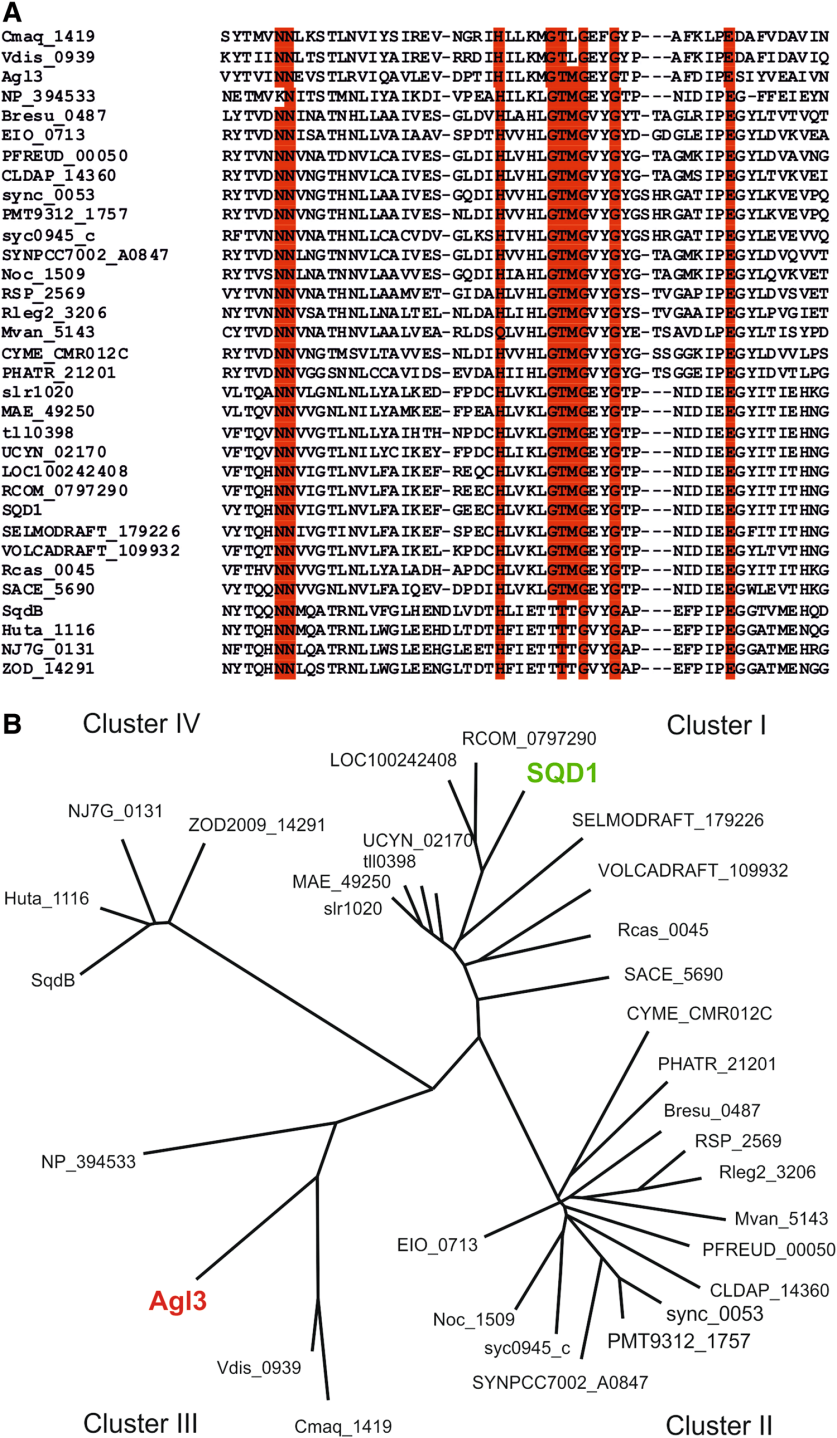
Homology alignment of amino acid sequences of UDP-sulfoquinovose synthases from plants, green algae, (cyano)bacteria, and archaea. **a** Alignment of selected regions of UDP-sulfoquinovose synthases showing the identical amino acids (*colored in red*) from *S. acidocaldarius*, plants, (cyano)bacteria, and selected species from thermophilic and extreme halophilic archaea. **b** Phylogenetic relationship of UDP-sulfoquinovose synthases from archaea, (cyano)bacteria, and plants. The designation of organisms and genes used for the construction of the phylogenetic tree is (*in alphabetical order*): *Arabidopsis thaliana*, SQD1; *Brevundimonas subvibrioides*, Bresu_0487; *Caldilinea aerophila*, CLDAP_14360; *Caldivirga* *maquilingensis*, Cmaq_1419; *Cyanidioschyzon merolae* strain 10D, CYME_CMR012C; *Cyanobacterium* UCYNA, UCYN_02170; *Haladaptatus paucihalophilus*, ZOD2009_14291; *Haloquadratum walsbyi*, SqdB; *Halorhabdus utahensis*, Huta_1116; *Ketogulonicigenium vulgare*, EIO_0713; *Microcystis aeruginosa* NIES-843, MAE_49250; *Mycobacterium vanbaalenii*, Mvan_5143; *Natrinema* sp. J7-2, NJ7G_0131; *Nitrosococcus* *oceani* ATCC 19707, Noc_1509; *Phaeodactylum tricornutum* CCAP 1055/1, PHATR_21201; *Propionibacterium freudenreichii*, PFREUD_00050; *Rhizobium leguminosarum* bv. *trifolii*, Rleg2_3206; *Rhodobacter sphaeroides*, RSP_2569; *Ricinus communis*, RCOM_0797290; *Roseiflexus castenholzii*, Rcas_0045; *Saccharopolyspora erythraea*, SACE_5690; *Selaginella moellendorffii*, SELMODRAFT_179226; *Sulfolobus acidocaldarius*, Agl3; *Synechococcus* sp., sync_0053; *Synechococcus* sp. PCC 7002, SYNPCC7002_A0847; *Synechocystis* sp. PCC 6803, slr1020; *Thermoplasma acidophilum*, NP_394533; *Thermosynechococcus elongatus*, tll0398; *Vitis vinifera*, LOC100242408; *Volvox carteri* f. *nagariensis*, VOLCADRAFT_109932; *Vulcanisaeta distributa*, Vdis_0939

As a starting point the phylogenetic relationship of UDP-sulfoquinovose synthases from archaea, (cyano)bacteria, and plants was analyzed. Agl3 from *S. acidocaldarius* is a 393-amino acid protein. Its alignment (http://www.ebi.ac.uk/Tools/msa/clustalw2) with 33 UDP-sulfoquinovose synthases available from the KEGG (http://www.genome.jp/dbgetbin/www_bget?ec:3.13.1.1) and NCBI (http://blast.ncbi.nlm.nih.gov/Blast.cgi) databases shows a high degree of sequence conservation, with 64 identical amino acids (Fig. 1a).

The phylogenetic analysis using ClustalW2 divides the UDP-sulfoquinovose synthases into four clusters (Fig. 1b). The (cyano)bacterial UDP-sulfoquinovose synthases are distributed in clusters I and II, with UDP-sulfoquinovose synthases from plants mainly localized in cluster II—SQD1 from *A. thaliana*, however, is localized in cluster I. In cluster III, Agl3 of *S. acidocaldarius* and UDP-sulfoquinovose synthases from other *Crenarchaeota* and hyperthermoacidophilic *Euryarchaeota* are localized. Apart from them are the UDP-sulfoquinovose synthases from halophilic *Euryarchaeota* (cluster IV). This cluster is more distant to the other three clusters, showing only 36 identical amino acids with other UDP-sulfoquinovose synthases. The enzyme of the halophilic archaea obviously evolved rather distant to those from plants, hyperthermoacidophilic archaea, and (cyano)bacteria. Among bacteria, the cyanobacterial UDP-sulfoquinovose synthases are predominant, but also other (non-photosynthetic) bacteria contain genes coding for this enzyme. The phylogenetic analysis showed that the occurrence of UDP-sulfoquinovose synthases in all domains of life obviously results from divergent evolution (Fig. 1b). Whether the categorization of the UDP-sulfoquinovose synthases into different clusters also reflects different reaction mechanisms is currently unknown but might be supported by observations made in the course of this work.

Since no crystal structure of Agl3 is available, we chose an approach for the prediction of functional epitopes similar to that used for SQD1 from *A. thaliana* (Essigmann et al. 1999) before a high-resolution crystal structure of this protein became available (Mulichak et al. 1999). The obtained model clearly shows high similarity of the overall fold of both proteins (for details see Supplemental Information, Figure S1).

### Point mutation studies of predicted critical amino acids in Agl3

**Fig. 2:**
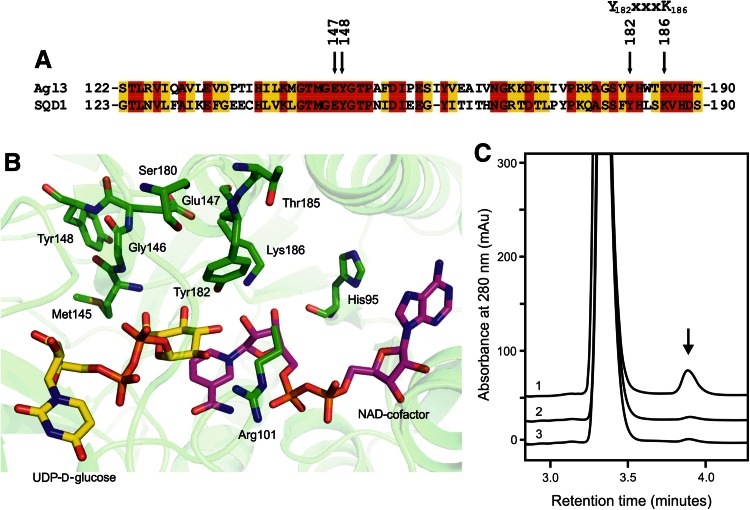
Partial amino acid comparison, close-up of the active site, and reactivity of wild-type and specific mutants of Agl3. **a** Selected region of the sequence alignment of Agl3 and SQD1 demonstrating a high degree of amino acid similarity of both enzymes. The positions of E147, Y148, and the characteristic Y-XXX-K motif of SDR proteins are indicated (Kavanagh et al. 2008). **b** Homology model of the active site of Agl3 with the critical amino acids drawn in *green sticks*, the NAD-cofactor in *pink* and the substrate in *yellow*. For clarity the backbone of residues 96–108 is not shown; (the figure was generated by pymol version 1.2r1). **c** In vitro conversion of UDP-d-glucose to UDP-d-glucose-5,6-*ene* (*arrow*) in absence of sulfite by the wild-type Agl3 (*graph 1*), Y148A mutant (*graph 2*), and E147A mutant (*graph 3*)

Upon comparison of the amino acid sequence of Agl3 with that of other UDP-sulfoquinovose synthases (Fig. 1a) including SQD1 (Fig. 2a) (Mulichak et al. 1999), potential residues of Agl3 were selected that could be involved in catalysis. Very similar to SQD1, our Agl3 model (Fig. 2b) comprises an N-terminal NAD^+^-binding domain with a highly conserved Rossmann fold and a C-terminal UDP-d-glucose binding domain. In SQD1, the catalytic residues were determined to be Thr145, Tyr182 and Lys186, with the latter two being present within the Y-XXX-K motif (Mulichak et al. 1999; Kavanagh et al. 2008). The corresponding amino acids in Agl3 are Thr144, Tyr182 and Lys186. From the respective alanine mutations (Tables 1, 2) the mutant T144A fully retained activity, whereas Ala replacement of His95, Arg101, Met145, Tyr182 and Lys186 (Fig. 2b) in Agl3 rendered the enzyme inactive. Both S180A and T185A mutant proteins showed impaired conversion of UDP-d-glucose to UDP-d-glucose-5,6-*ene* (Tables 1, 2).

**Table 1:** Alanine mutations of selected amino acids of Agl3: used primers, primer sequence, target mutation and overall mutant enzyme activity

Primer name	Primer sequence	Target mutation	Mutant enzyme activity^a^
Sqsyn1-Forward-H95A	3′-GCCATAGTGGCTTTCGCTGAG-5′	Histidine 95	
Sqsyn2-Reverse-H95A	3′-CTCAGCGAAAGCCACTATGGC-5′	Histidine 95	−
Sqsyn3-Forward-R101A	3′-GCTGAGCAGGCTTCTGCTCCG-5′	Arginine 101	
Sqsyn4-Reverse-R101A	3′-CGGAGCAGAAGCCTGCTCAGC-5′	Arginine 101	−
Sqsyn5-Forward-T144A	3′-AAGATGGGTGCTATGGGTGAG-5′	Threonine 144	
Sqsyn6-Reverse-T144A	3′-CTCACCCATAGCACCCATCTT-5′	Threonine 144	++
Sqsyn7-Forward-M145A	3′-ATGGGTACCGCTGGTGAGTAT-5′	Methionine 145	
Sqsyn8-Reverse-M145A	3′-ATACTCACCAGCGGTACCCAT-5′	Methionine 145	−
Sqsyn19-Forward-E147A	3′-ACCATGGGTGCCTATGGAACACCT-5′	Glutamic acid 147	
Sqsyn20-Reverse-E147A	3′-AGGTGTTCCATAGGCACCCATGGT-5′	Glutamic acid 147	(±)
Sqsyn21-Forward-Y148A	3′-CATGGGTGAGGCTGGAACACCT-5′	Tyrosine 148	
Sqsyn22-Reverse-Y148A	3′-AGGTGTTCCAGCCTCACCCATG-5′	Tyrosine 148	(±)
Sqsyn9-Forward-S180A	3′-GCGGGTGCTGTTTATCAC-5′	Serine 180	
Sqsyn10-Reverse-S180A	3′-GTGATAAACAGCACCCGC-5′	Serine 180	(+)
Sqsyn11-Forward-Y182A	3′-GGTTCTGTTGCTCACTGGACT-5′	Tyrosine 182	
Sqsyn12-Reverse-Y182A	3′-AGTCCAGTGAGCAACAGAACC-5′	Tyrosine 182	−
Sqsyn15-Forward-T185A	3′-TATCACTGGGCTAAGGTTCAT-5′	Threonine 185	
Sqsyn16-Reverse-T185A	3′-ATGAACCTTAGCCCAGTGATA-5′	Threonine 185	(+)
Sqsyn17-Forward-K186A	3′-CACTGGACTGCTGTTCATGAT-5′	Lysine 186	
Sqsyn18-Reverse-K186A	3′-ATCATGAACAGCAGTCCAGTG-5′	Lysine 186	–

^a^Qualitative determination: ++ strongly active, + active, (+) lesser active, (±) less active, – inactive protein

**Table 2:** Alanine mutations of selected amino acids of Agl3: enzymatic characterization of wild-type Agl3 and the mutants E147A and Y148A

Enzyme protein	Total reaction volume (µl)	Amount of enzyme (mg)	Total yield (Units^a^)	Specific activity (U mg^−1^ of protein)	Total activity (%)
Wild-type Agl3	200	0.121	0.00673	0.0556	100
E147A mutant	200	0.135	0.00094	0.0069	12.4
Y148A	200	0.129	0.00109	0.0084	15

^a^One unit is defined as micromole of product (UDP-d-glucose-5,6-*ene*) produced per milligrams of enzyme protein in 30 min

**Fig. 3:**
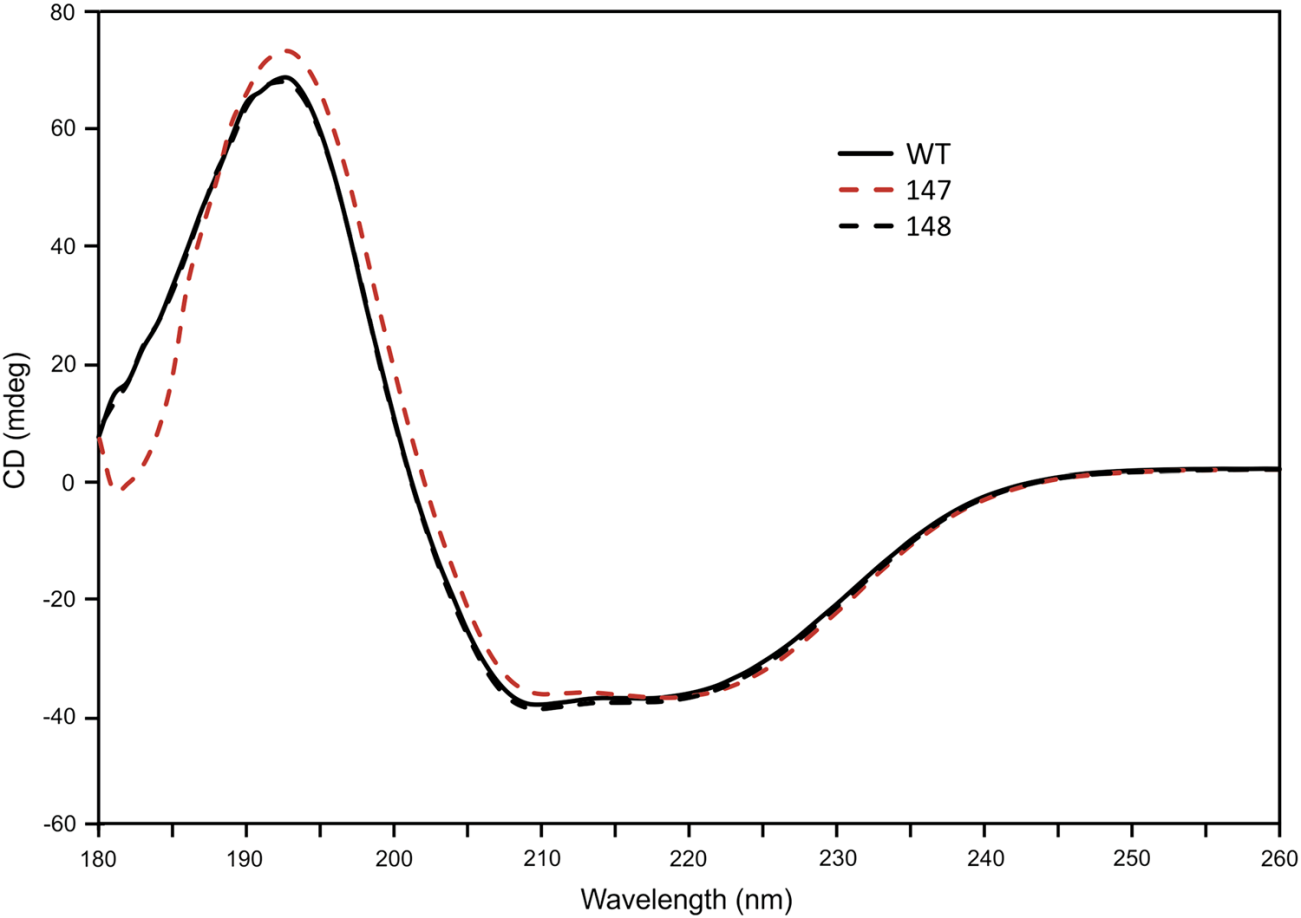
Far-UV CD spectra of 7 μM wild-type Agl3 and the mutants E147A and Y148A in 20 mM phosphate buffer, pH 7.0

Since most of the selected mutants were inactive and did not allow any prediction of a possible reaction pathway, additional mutations were introduced which open the possibility of a dehydratase mechanism for Agl3 (Gross et al. 2001; Hegeman et al. 2001). It was previously shown that in the dehydration reaction of dTDP-d-glucose-4,6-dehydratase (Allard et al. 2002) and GDP-d-mannose-4,6-dehydratase (Somoza et al. 2000) the two active site amino acids tyrosine and glutamic acid are crucial for the acid-catalyzed release of the hydroxyl group at C-6 of the substrate. According to our active site model of Agl3 (Fig. 2b), these amino acids could be represented by Tyr148 and Glu147 in Agl3, supporting a possible UDP-d-glucose dehydratase activity for UDP-sulfoquinovose biosynthesis. The respective alanine mutants (Tables 1, 2) showed strongly impaired conversion of UDP-d-glucose to UDP-d-glucose-5,6-*ene* (Fig. 2c) in the absence of sulfite, supporting their important roles in the catalytic activity of Agl3 and the assumed dehydratase-like function. To ensure that the observed decrease in activity was not a result of protein instability, far-UV circular dichroism spectra of wild-type Agl3 and the variants E147A and Y148A were recorded. Figure 3 demonstrates the mainly α-helical structure of Agl3 as well as the almost identical overall secondary structure composition of the three mutant proteins.

In the homology model of the putative active site of Agl3, the residues Glu147 and Tyr148 are at a distance of >5 Å away from UDP-d-glucose (Fig. 2b). This suggests that either a conformational change of the G-XX-G-XX-G motif must occur to facilitate the reaction or UDP-d-glucose needs to change its orientation to favorably interact with these residues. Overall, UDP-sulfoquinovose synthases contain several highly conserved glycine and proline residues, suggesting a highly conserved structural mode of switching Agl3 from an inactive to an active conformation.

### Catalytic conversion of UDP-d-glucose to UDP-d-glucose-5,6-*ene* in the absence of a sulfur donor

**Fig. 4:**
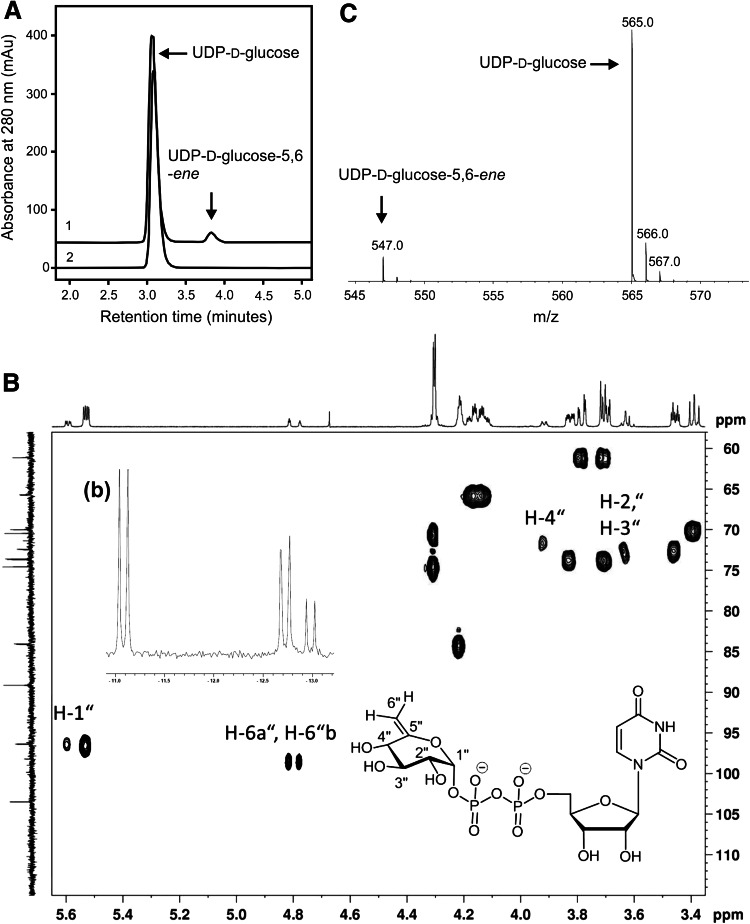
Conversion of UDP-d-glucose by Agl3 in the absence of sulfite (**a**) RP-HPLC analysis of the conversion of UDP-d-glucose to the UDP-d-glucose-5,6-*ene* intermediate (*arrow* at 3.8 min) in the presence of Agl3 (*graph 1*). In the negative control experiment (absence of Agl3; *graph 2*), no conversion was observed. **b** NMR expansion plot of an edHSQC spectrum of the mixture of UDP-d-glucose (major component) and UDP-d-glucose-5,6-*ene* (minor component from the enzymatic conversion). The inset (**b**) shows an expansion plot of the ^31^P NMR spectrum of the mixture displaying the intact diphosphate linkages of both compounds. **c** ESI–MS analysis of UDP-d-glucose-5,6-*ene*

Previously, we demonstrated that recombinant Agl3 from *S. acidocaldarius* is active in vitro, converting UDP-d-glucose and sulfite to UDP-sulfoquinovose in a yield of approximately 10 % (Meyer et al. 2011). Here, we show that in the absence of sulfite, the in vitro conversion of UDP-d-glucose results in the formation of UDP-d-glucose-5,6-*ene* (Fig. 4). This new reaction product eluted at a retention time of 3.8 min in the RP-HPLC experiment (Fig. 4a). The Agl3-catalyzed conversion of UDP-d-glucose was further investigated by NMR spectroscopy and ESI–MS (Fig. 4b, c). Supplementation of the reaction mixture with NAD^+^, NADH or FAD to a final concentration of 1 mM, each, did not result in a higher catalytic conversion of UDP-d-glucose, as was concluded from the yield and retention time of the reaction product upon RP-HPLC separation (not shown). The ^1^H NMR spectrum of that sample revealed the signals of non-reacted UDP-d-glucose (verified by using an UDP-d-glucose standard) and of a second compound in a ratio of 2.8:1, as was deduced from the integrated signals of the respective anomeric protons (at 5.5 and 5.6 ppm, respectively). The minor, slightly low-field shifted anomeric proton of the second compound displayed a heteronuclear coupling to ^31^P, and the presence of an intact diphosphate unit was confirmed by the chemical shifts in the ^31^P NMR spectrum (Fig. 4b, inset). Despite overlapping signals from the UDP-component of UDP-d-glucose and the reaction product, the remaining signals could be fully assigned using COSY, TOCSY, edHSQC and HMBC data. Thus, structural proof for the presence of an exocyclic double bond was obtained from the edited HSQC spectrum (Fig. 4b). Two low-field shifted ^1^H NMR signals were observed at 4.81 and 4.78 ppm with a correlation to a ^13^C NMR signal at 98.5 ppm. Both protons displayed small values for the geminal coupling constant as well as allylic spin–spin couplings to H-4″. The latter signal revealed a large coupling constant confirming a *trans*-orientation relative to H-3″, in agreement with a *gluco*-configuration. Final support for the proposed UDP-d-glucose-5,6-*ene* structure was derived from HMBC-correlations of H-4″ as well as H-1″ to a ^13^C NMR signal at 155.3 ppm, which are in full agreement with the partial structure of an exocyclic enol ether (Table 3). Based on these data, the structure of the enzymatic reaction product could be unambiguously determined as UDP 6-deoxy-d-*xylo*-hex-5-enose (UDP-d-glucose-5,6-*ene*). Signals for keto or hydrated keto groups as well as signals for deuterated UDP-d-glucose in the product mixture could not be detected within the detection limit of the NMR instrument. Also, in situ monitoring of the enzymatic reaction in an NMR tube for 5 h at 333 K (60 °C) revealed a slow formation of the UDP-d-glucose-5,6-*ene* product but did not provide direct evidence of a 4-keto intermediate (Li et al. 2014).

**Table 3:** NMR data of UDP-d-glucose-5,6-*ene*

Atom	^1^H (ppm)	*J* (Hz)	^13^C (ppm)	HMBC correlation
1″	5.59	3.0/7.9	96.4	155.3
2″	3.63	n.d.	72.1	–
3″	3.62	9.7/9.4	72.7	–
4″	3.91	9.4/2 × 2.0	71.6	–
5″	–	–	155.3	–
6″a	4.81	1.7/1.9	98.5	155.3
6″b	4.78	1.6/2.1	98.5	–
1′	5.90	n.d.	89.3	–
2′	4.31–4.29	n.d.	74.6	–
3′	4.31–4.29	n.d.	70.7	–
4′	4.22–4.20	n.d.	84.3	–
5a′/5′b	4.18–4.11	n.d.	65.8	–
2	–	–	n.d.	–
4	–	–	n.d.	–
5	5.90	–	103.7	–
6	7.88	8.1	142.9	–
	^31^P (ppm)			
5′-linked	−11.08	20.5	–	–
1″-linked	−12.98	20.5	–	–

*n.d.* not determined

The MS-spectrum of the reaction mixture of UDP-d-glucose conversion showed a major peak at 565.0 u (corresponding to the substrate), accompanied by two smaller peaks at 566.0 and 567.0 u, respectively, and one further, though even smaller, peak at 547.0 u showing the same pattern of accompanying peaks, each one unit apart, as seen with the major peak, but with much less intensity (Fig. 4c).

### Formation of UDP-d-glucose-5,6-*ene* in the course of UDP-sulfoquinovose biosynthesis

**Fig. 5:**
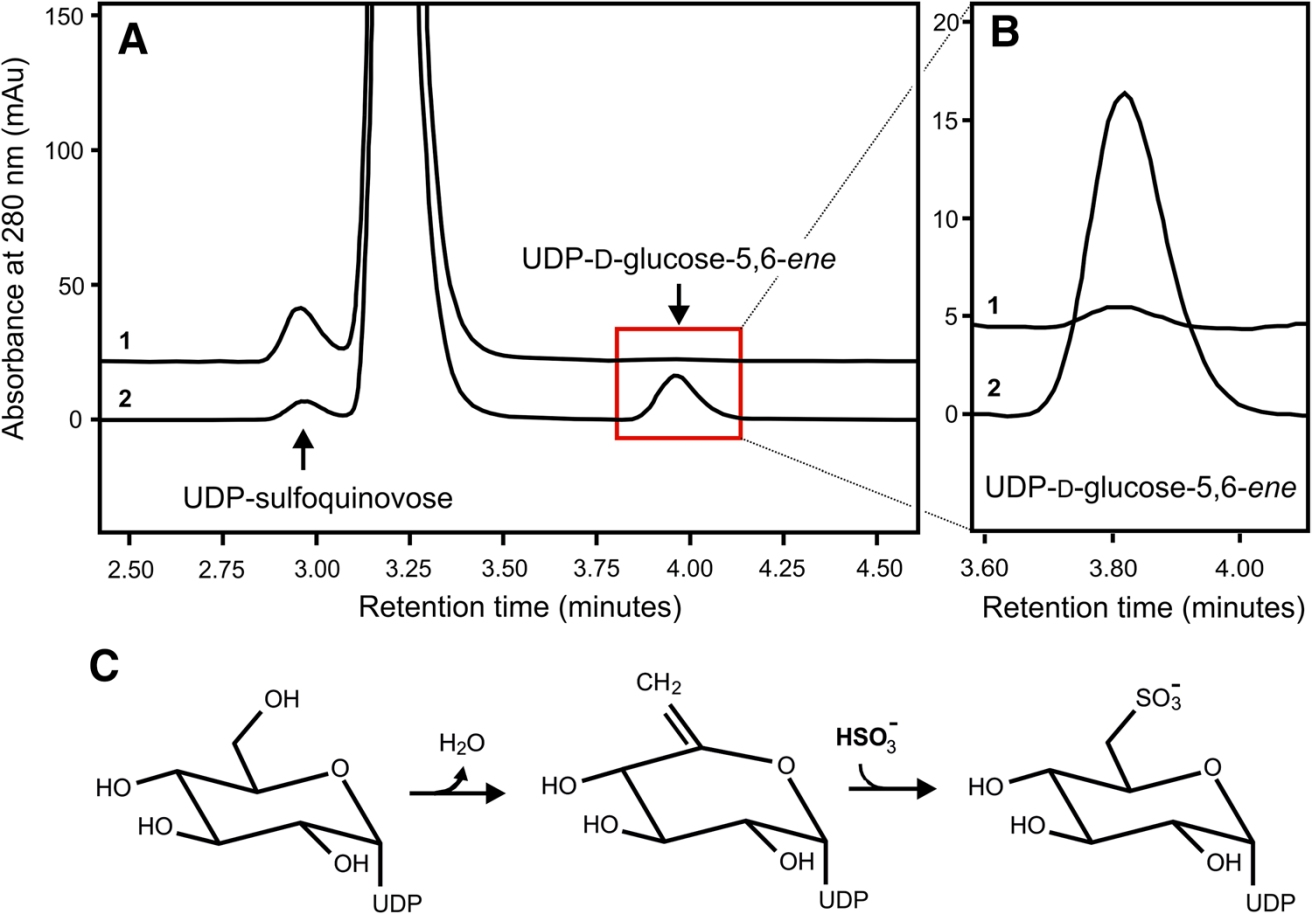
HPLC analysis of the conversion of UDP-d-glucose and sulfite to UDP-sulfoquinovose by Agl3 (**a**) UDP-sulfoquinovose biosynthesis was analyzed under sulfite-saturated (*graph 1*) and unsaturated conditions (*graph 2*). **b** The UDP-d-glucose-5,6-*ene* is present at sulfite-saturated UDP-sulfoquinovose biosynthesis (*graph 1*; only trace amounts), and under unsaturated conditions (*graph 2*). **c** dehydration of UDP-d-glucose is a key catalytic activity of Agl3

To investigate the formation of UDP-d-glucose-5,6-*ene* (Fig. 5) in detail, the RP-HPLC profile of the conversion of 3 mM UDP-d-glucose to UDP-sulfoquinovose was compared at high (3 mM; Fig. 5a, graph 1), and low sulfite concentration (0.1 mM; Fig. 5a, graph 2). At 3 mM sulfite, Agl3 rapidly converted UDP-d-glucose to UDP-sulfoquinovose (Fig. 5a, graph 1), with complete product formation as indicated by the lack of detectable UDP-d-glucose-5,6-*ene*. In contrast, at 0.1 mM sulfite, a considerable accumulation of UDP-d-glucose-5,6-*ene* occurred in the reaction mixture (Fig. 5b, graph 2). Under sulfite-saturated conditions, UDP-d-glucose is first converted to UDP-d-glucose-5,6-*ene*, followed by conversion into UDP-sulfoquinovose (Fig. 5c). Based on the kinetics of UDP-sulfoquinovose biosynthesis, in combination with the accumulation of UDP-d-glucose-5,6-*ene* in the absence of sulfite, we conclude that conversion of UDP-d-glucose to UDP-sulfoquinovose by Agl3 is a two-step reaction with UDP-d-glucose-5,6-*ene* being a crucial reaction intermediate of this pathway (Figs. 4, 5). However, due to the available analytical tools in our laboratory, we were only able to isolate and unambiguously characterize starting and end points of these reactions. The appearance of UDP-d-glucose-5,6-*ene* is taken as an additional indication of a dehydration step being included in the complex Agl3 reaction mechanism.

### Preliminary investigations of the dehydration mechanism of Agl3 and role of the NAD-cofactor

Monitoring of the conversion of UDP-d-glucose is challenging, because of the low in vitro activity of Agl3 and the fact that the absorption spectra of UDP-d-glucose, UDP-d-glucose-5,6-*ene* and UDP-sulfoquinovose are identical. Supplementing the reaction mixture with NAD^+^ during Agl3 activity measurement did not lead to accumulation of NADH (monitored at 340 nm). This is consistent with our observation that supplementing with NAD^+^ does not increase the catalytic activity of Agl3 (data not shown) and indicative of a tight binding of NAD^+^ to the polypeptide matrix of Agl3 and no exchange with the medium.

**Fig. 6:**
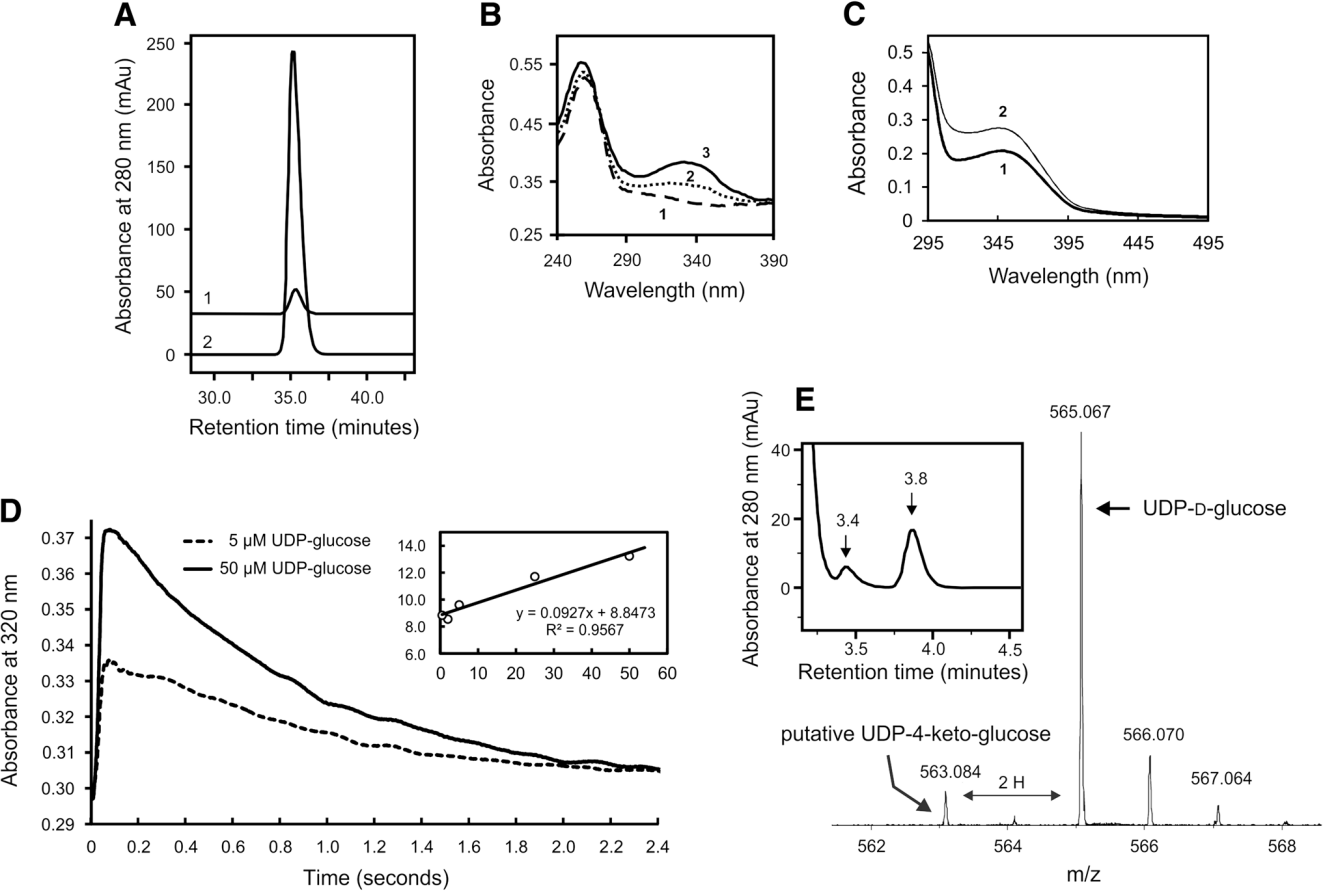
Cofactor analysis and kinetics of NAD^+^ reduction of UDP-d-glucose. **a** RP-HPLC analysis of NAD^+^ extracted from Agl3 in oxidized form (*graph 1*) compared to NAD^+^ reference (*graph 2*). **b** Absorption spectra of the extracted cofactor in the fully oxidized (NAD^+^; spectrum 1) and reduced (NADH) state. Reduction was mediated by glucose dehydrogenase and glucose. Spectra were recorded after 2 min (spectrum 2) and 5 min (spectrum 3). **c** Absorption spectrum of Agl3 in the resting state (spectrum 1) suggesting a mixed oxidation state of the tightly bound cofactor. Spectrum 2 is formed immediately after addition of 50 μM UDP-d-glucose. **d** Stopped-flow analysis of the reduction of the bound NAD-cofactor by UDP-d-glucose. Two representative time traces for the reaction between 2 μM Agl3 and 5 or 50 μM UDP-d-glucose are depicted. The first rapid increase at 320 nm depends on the UDP-d-glucose concentration. The *inset* shows the corresponding plot of the *k*_obs_ values for this reaction versus the concentration of the electron donor. **e** RP-HPLC analysis and MS analysis of a new reaction product at 3.4 min retention time (HPLC graph) and a molecular mass of 563 gram/mole (mass spectrum), extracted during the conversion of UDP-d-glucose to UDP-d-glucose-5,6-*ene* by chloroform in the absence of sulfite and at an Agl3 concentration of 2.7 mg ml^−1^

The nature of the tightly bound prosthetic group in Agl3 was investigated after chloroform extraction (Fig. 6a). RP-HPLC analysis confirmed the presence of NAD^+^ in Agl3 by comparison with authentic NAD^+^ reference material (Fig. 6a, graph 2). Fractionation and spectrophotometric analysis of the extracted cofactor showed a typical NAD^+^ absorption spectrum in the oxidized state (Fig. 6b, spectrum 1). Subsequently, the nature of the extracted NAD-cofactor was confirmed as NAD^+^ in an in vitro biocatalytic experiment (Fig. 6b, spectra 2 and 3) by adding trace amounts of NAD(P)-dependent glucose dehydrogenase and glucose (1 mM final concentration) to the extracted cofactor. After 2 min of incubation at room temperature, NAD^+^ was reduced, evidenced by an increase of absorbance (Fig. 6b, spectrum 2), which was even further pronounced after 3 min of incubation (Fig. 6b, spectrum 3), since glucose dehydrogenase converted glucose to d-glucono-1,5-lactone thereby transferring the electrons to NAD^+^ and yielding NADH.

Agl3 was, in any case, tested as a holo enzyme because all attempts to prepare NAD-free apo-Agl3 failed. Extensive dialysis (2 days) did not remove the bound NAD-cofactor; the absorption spectrum of Agl3 at 340 nm still showed the presence of cofactor inside the enzyme. Dialysis for longer time led to precipitation of Agl3 and complete loss of activity (not shown).

Since determination of the NAD-dependent activity of Agl3 could not be monitored at steady-state conditions, the pre-steady-state kinetics of the reaction was investigated by stopped-flow experiments. We probed whether the bound cofactor is able to accept electrons from UDP-d-glucose at 65 °C, mimicking the natural growth temperature of *S. acidocaldarius*. As depicted in Fig. 6c (spectrum 1), in the resting state of recombinant Ag13 (2 µM), the spectrum of the pyridine nucleotide cofactor suggests the occurrence of a mixture of NAD^+^ and NADH. Upon addition of 50 µM UDP-d-glucose there was a rapid formation of fully reduced NADH (Fig. 6c, spectrum 2) followed by a slow re-oxidation to the mixed NAD^+^/NADH state. The corresponding time traces of this biphasic reaction are shown in Fig. 6d for 5 and 50 µM UDP-d-glucose, respectively. The first rapid absorbance increase at 320 nm is dependent on the concentration of the electron donor. Upon fitting this phase by a single-exponential function, *k*_obs_ values could be estimated. From the slope of the linear plot of *k*_obs_ values versus the concentration of UDP-d-glucose, an apparent bimolecular rate constant of ~9.3 × 10^4^ M^−1^ s^−1^ could be estimated at 65 °C and pH 7.0. The high intercept of 8.8 s^−1^ suggests that transiently formed NADH becomes re-oxidized. After about 3 s the mixed NAD^+^/NADH state of the enzyme sample was established again (Fig. 6d). The second slower phase (i.e., absorbance decrease at 320 nm) did not depend on the sugar concentration. Consistent with the literature (Mulichak et al. 1999, 2002; Hegeman et al. 2001), we propose that the conversion of UDP-d-glucose to UDP-d-glucose-5,6-*ene* by Agl3 is initiated by oxidation of the hydroxyl group at C-4 of the substrate by NAD^+^, leading to NADH. This is first demonstration of the rapid electron transfer between UDP-d-glucose and NAD^+^ in a UDP-sulfoquinovose synthase (Fig. 6c, d).

The oxidation of UDP-d-glucose was accomplished at high enzyme concentration (2.7 mg ml^−1^). Chloroform was directly added to the reaction mixture, resulting in an instant unfolding of Agl3 and release of the reaction product into the water phase. RP-HPLC analysis of the latter showed the presence of the new compound at 3.4 min retention time (~40 pmol) (Fig. 6e), eluting between the substrate UDP-d-glucose (3.2 min) and UDP-d-glucose-5,6-*ene* (3.8 min). MS analysis of the new reaction product yielded a molecular mass of 563.084 u (ESI–MS analysis in the negative mode; Fig. 6e). This value obviously corresponds to the molecular mass of oxidized UDP-d-glucose. The accumulation of the new product was only detectable at high Agl3 concentration. The putative keto-compound remained tightly associated with the active site of Agl3 during substrate turnover, and could be extracted only by instantaneous chloroform-induced unfolding of Agl3 during the catalytic conversion of UDP-d-glucose to UDP-d-glucose-5,6-*ene* (Sporty et al. 2008). Because of the fast rate of conversion, the accumulation of the putative keto-compound is very low, thus preventing structural analysis by NMR. The existence of a 4-keto product in the reaction pathway of different SDR enzymes was shown in plants (Pugh et al. 1995; Mulichak et al. 1999) and for the model enzymes GalE (Thoden et al. 1996a, b) and RmlB (Allard et al. 2002). Currently it is not possible to continuously monitor every step of the formation of UDP-sulfoquinovose and thus, the overall mechanism of this bi-substrate enzyme is not yet fully understood.

### Conversion of UDP-d-glucose by Agl3, analyzed after deuterium labeling

**Fig. 7:**
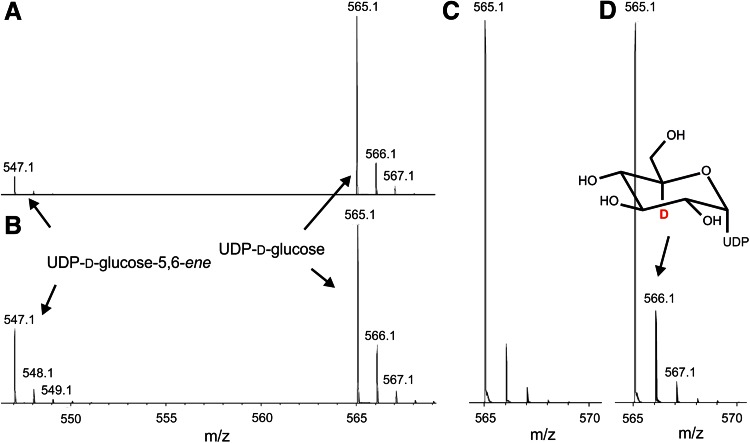
Conversion of UDP-d-glucose to UDP-d-glucose-5,6-*ene* by Agl3 at an excess of deuterium. **a** Conversion of UDP-d-glucose to UDP-d-glucose-5,6-*ene* reaching an equilibrium reaction at steady-state level. **b** MS analysis of the conversion of UDP-d-glucose to UDP-d-glucose-5,6-*ene* in H_2_O-phosphate buffer compared to (**c**) its conversion in D_2_O-phosphate buffer. **d** Natural isotope distribution pattern of UDP-d-glucose measured by ESI–MS in the negative mode in H_2_O-phosphate

The conversion of UDP-d-glucose to UDP-d-glucose-5,6-*ene* at an excess of D_2_O (Fig. 7) indicated enolization and locking of deuterium at C-5 of UDP-d-glucose (Gross et al. 2000). For this approach, purified Agl3 was dialyzed against 20 mM phosphate buffer of pD 6.4. Substrate conversion in D_2_O-phosphate buffer was repeated several times to obtain sufficient material for the subsequent ESI–MS analysis. As a result, 15 % of UDP-d-glucose were labeled with one deuterium atom, with the ratio of isotope distribution in UDP-d-glucose-5,6-*ene* after enzymatic conversion in D_2_O-phosphate remaining unchanged compared to the substrate (Fig. 7; Table 4). This indicated that the altered isotope distribution was specific for UDP-d-glucose and not the result of a deuterium contamination. Additionally, the stability of UDP-d-glucose in D_2_O-phosphate buffer was tested, confirming that in the absence of Agl3, UDP-d-glucose remained stable and unlabeled (not shown). Interestingly, the catalytic conversion of UDP-d-glucose to UDP-d-glucose-5,6-*ene* in D_2_O-phosphate buffer increased to nearly 50 % compared to the conversion in H_2_O (~10 %) (Fig. 7a, b).

**Table 4:** Ratio of isotope distribution of UDP-d-glucose and UDP-d-glucose-5,6-*ene* from enzymatic conversion performed in H_2_O and excess of D_2_O

*m*/*z*	Ratio of isotope distribution of UDP-d-glucose-5,6-*ene* from enzymatic conversion in excess D_2_O	Ratio of isotope distribution of UDP-d-glucose-5,6-*ene* from enzymatic conversion in H_2_O
547	1	1
548	0.185	0.195
549	0.053	0.053

*m*/*z*	Ratio of isotope distribution of UDP-d-glucose from enzymatic conversion in excess D_2_O	Ratio of isotope distribution of UDP-d-glucose from enzymatic conversion in H_2_O
565	1	1
566	0.33	0.189
567	0.08	0.053

**Fig. 8:**
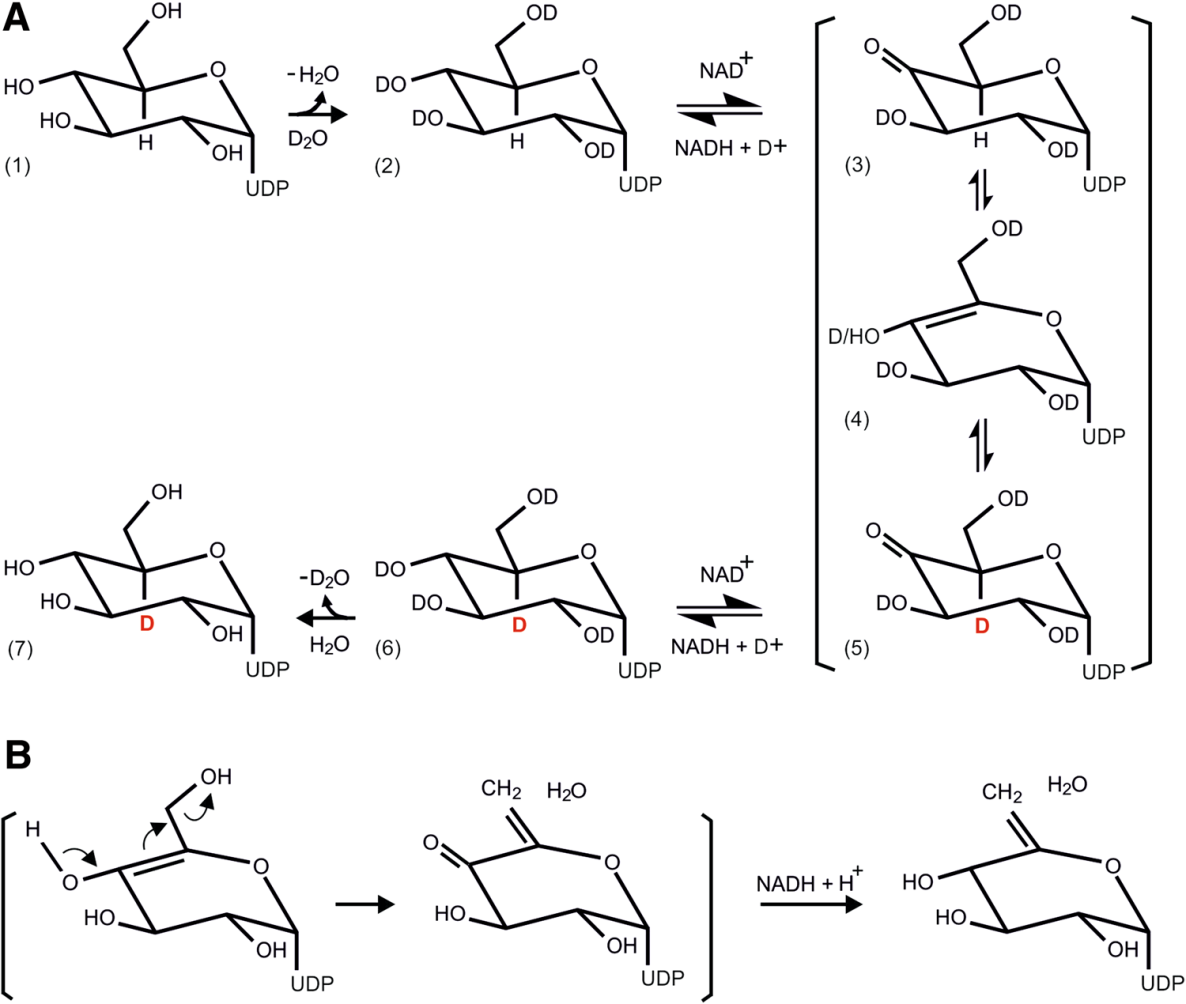
Proposed mechanism for the conversion of UDP-d-glucose to UDP-d-glucose-5,6-*ene* (**a**) NAD-dependent oxidation of UDP-d-glucose at C-4 (structure 3), enolisation of UDP-4-keto-glucose (structure 4) and locking of deuterium at C-5 by the enolisation process at the active site of Agl3 (structures 5–7). **b** Proposed NAD-dependent dehydration of the enol-conformation of UDP-4-keto-glucose eventually resulting in UDP-d-glucose-5,6-*ene*. 160 × 135 mm (300 × 300 DPI)

**Fig. 9:**
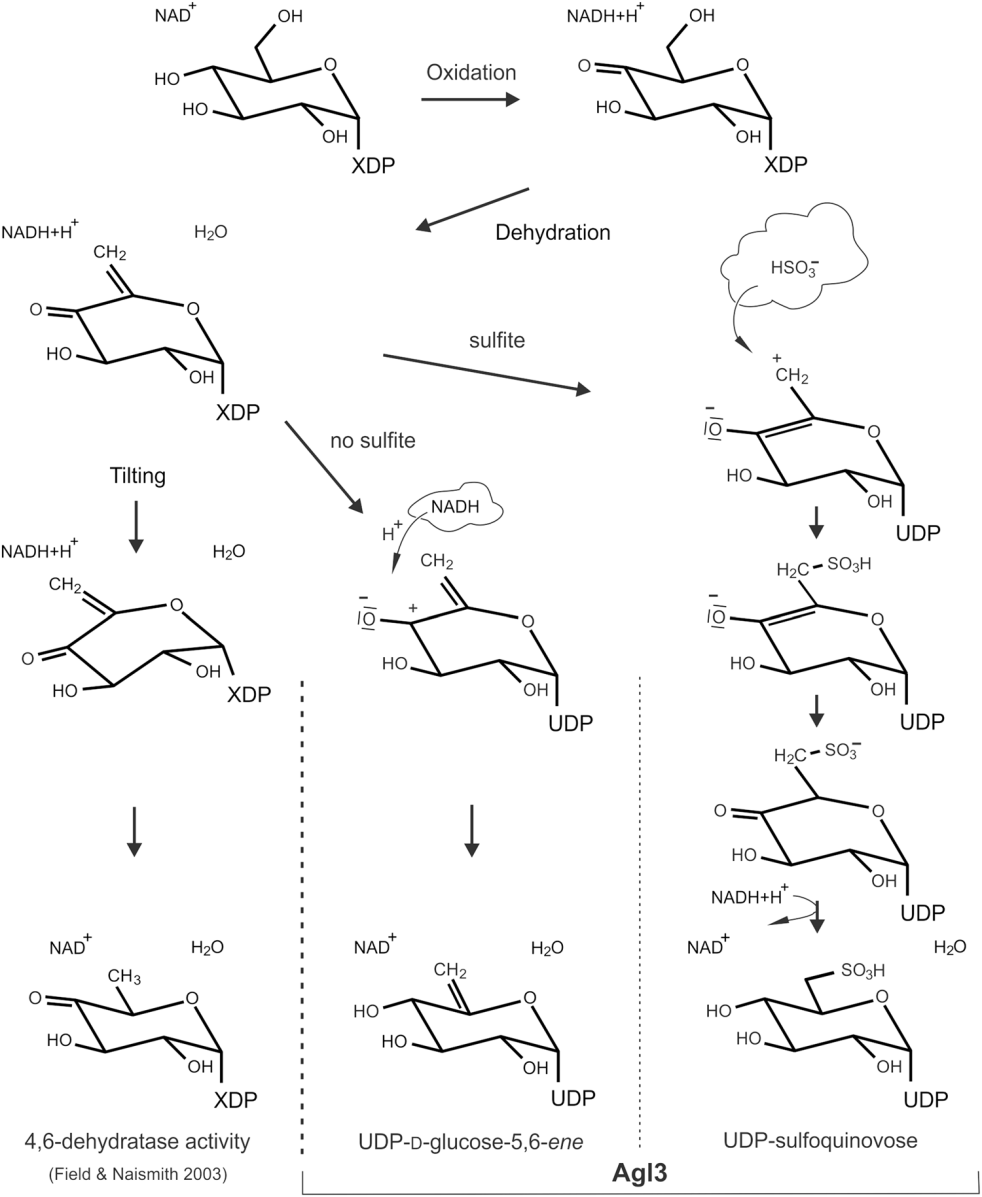
Comparison of reaction mechanisms of classical 4,6-dehydratases (Field and Naismith 2003) and the proposed reaction mechanisms of Agl3 in the absence and presence of sulfite resulting either in the formation of UDP-d-glucose-5,6-*ene* or UDP-sulfoquinovose. 161 × 197 mm (300 × 300 DPI)

The assumed keto-enol tautomeric equilibrium after oxidation of the OH group at C-4 of UDP-d-glucose was attempted to be shown by deuterium labeling of the substrate at the active site of Agl3 (Gross et al. 2000) (Fig. 8). This inter-conversion requires the release of the hydrogen at C-5 of the putative UDP-4-keto-glucose (Fig. 8a, structure 3) and subsequent formation of a double bond between C-4 and C-5 (Fig. 8a, structure 4). The inter-conversion of the putative UDP-4-keto-glucose and its enol form directly at the active site of Agl3 was traced by locking a deuterium atom at the C-5 position of the substrate by the enolization process (Fig. 8a, structures 4, 5), and labeling of UDP-d-glucose with deuterium when the conversion of UDP-d-glucose by Agl3 was performed at an excess of D_2_O (Fig. 8a, structure 6). Labeling of C-5 with deuterium (e.g. C-5 of dTDP-d-glucose of RmlB) has been proposed for nucleotide-activated glucose intermediates involving keto-formation at C-4 followed by enolization between C-4 and C-5 (Gross et al. 2000). The observed labeling of UDP-d-glucose with deuterium (Fig. 8a, structures 6, 7) also suggests that dehydration of UDP-d-glucose occurs only from the enol form of UDP-4-keto-glucose (Fig. 8a, structure 4). This does explain why UDP-d-glucose-5,6-*ene* at C-5 could not be labeled with deuterium (Fig. 8b; Table 4). Deuterium is cleaved off from UDP-4-keto-glucose at the active site of Agl3 prior to its dehydration (Fig. 8a, inter-conversion of structure 5 to 4). Thus, dehydration of UDP-4-keto-glucose is proposed to occur through the enol form (Fig. 8b), followed by the transfer of hydride to C-4. The assumed hydride transfer diverts here from the common dehydratase pathway, where hydride transfer to C-6 has been proposed (Field and Naismith 2003). This step might be accomplished without the suggested sugar rotation and still keep C-4 in an optimal position for hydride transfer. It would allow sulfite addition to an enone for subsequent UDP-sulfoquinovose biosynthesis (Fig. 9) but we cannot structurally prove our proposal because of the current lack of a crystal structure of Agl3.

An interesting observation concerns the nearly 50 % increase of the catalytic conversion of UDP-d-glucose to UDP-d-glucose-5,6-*ene* in D_2_O-phosphate buffer when compared to the conversion in H_2_O-phosphate buffer (Fig. 8). There is sufficient volume for a significant amount of water molecules to be present at the active site of Agl3 (see supplemental Figure S1), which, through H-bridging, stabilizes the interaction between the crucial residues of Agl3 and UDP-d-glucose. D_2_O has a stronger dipole moment than H_2_O. Therefore, stronger H-bridge interaction inside of Agl3 in D_2_O-phosphate buffer most likely leads to increased stability of the active site of Agl3 and increased catalytic activity of the enzyme.

### Evolutionary relationship between UDP-sulfoquinovose synthases, including Agl3

UDP-sulfoquinovose synthases belong to the SDR subfamily of oxidoreductases containing a conserved cofactor-binding Rossmann-fold domain (Kavanagh et al. 2008). In the current study, we confirmed that the reaction mechanism of Agl3 of *S. acidocaldarius* follows the general mechanism of this subfamily. This proof is particularly important because of the possible distant evolutionary relationship between Agl3 and SQD1 from *A. thaliana* (Fig. 1a).

If the reaction mechanisms of Agl3 and SQD1 are indeed different, other crucial amino acids than those identified for SQD1 should be involved in the catalytic reaction. This implicates presumably a dehydratase reaction in Agl3 (Allard et al. 2002; Field and Naismith 2003). The respective catalytic tyrosine residue in Agl3 could be Tyr148 (Figs. 1, 2a, b). It might function as active site base and accomplish—in concert with Glu147—the dehydration step (Allard et al. 2002). With the RmlB enzyme from *Salmonella enterica* sv. *Typhimurium* it was shown that the C-4 of the substrate was at the optimal position for initial hydride abstraction (Allard et al. 2002). The results obtained with Agl3 are in agreement with this reaction step (Fig. 9), in which NAD^+^ initially oxidizes glucosyl C-4 of dTDP-glucose to dTDP-4-keto-glucose, leaving the NAD-cofactor reduced. Next, water is eliminated between C-5 and C-6 of dTDP-4-keto-glucose to form dTDP-4-keto-glucose-5,6-*ene*. Hydride transfer from NADH to C-6 of dTDP-4-keto-glucose-5,6-*ene* regenerates NAD^+^ and produces the product dTDP-4-keto-6-deoxyglucose (Gross et al. 2000). It was proposed that water remains bound to the protein and sugar rotation around the glycosidic linkage creates no steric clash but should move C-6 to an appropriate location for accepting the hydride (Field and Naismith 2003).

The reaction pathway of the UDP-sulfoquinovose synthase Agl3 of S. *acidocaldarius* obviously follows the general pathway described for dehydratases (Field and Naismith 2003), but seems to be modified at the final step (Fig. 9). Based on our data we assume the final hydride transfer to occur to C-4 rather than C-6 of the enone form of UDP-4-keto-glucose, leaving this carbon accessible for the subsequent addition of sulfite (Fig. 9). Conclusive information about the actual reaction mechanism of Agl3 is expected from 3D crystallization experiments which are currently in progress.

## Procedures

### Agl3 expression and point mutation analysis

Recombinant production and nickel affinity purification of hexa-histidine-tagged Agl3 from *S.* *acidocaldarius* were performed as described previously (Meyer et al. 2011). Point mutations were performed by overlap-extension PCR. Ten amino acid positions in Agl3 were selected on the basis of critical amino acids either from epimerases or dehydratases (Mulichak et al. 1999; Field and Naismith 2003). Targeted amino acids in Agl3 along with the primers carrying the alanine codon for replacement of the selected codon are shown in Table 1, 2. First, *agl3* was cloned into pET30a (Novagen), using the primer pair 3′-CCCCCCCATATGAGGATTCTAGTACTAGGAATT-5′/3′-CCCCCCCTCGAGACCACCCGCACCACCTCTTACTCTTTTAACGTATTGTGGTTT-5′. The first phase of overlap-extension PCR was performed with 7 ng of pET30_Agl3 as a template, one unit of Phusion polymerase (Fermentas), 10 mM of dNTP mix and amplification conditions as follows: one cycle at 94 °C for 4 min, followed by 30 cycles at 94 °C for 30 s, each, for denaturation, and annealing at 55 °C for 30 s, extension/elongation at 72 °C for 90 s, and one final elongation cycle at 72 °C for 5 min. The amplification products were purified using the gel extraction kit from Fermentas and used as templates (approximately 5 ng, each) for the second phase of the overlap-extension PCR using identical amplification conditions. The amplification products were purified (see above) and cloned into pET30a via the *Nde*I and *Xho*I restriction sites (Meyer et al. 2011). The point mutations were confirmed by single-run sequencing (LGC genomics); the Agl3 proteins carrying the different alanine mutations were expressed in *E.* *coli* BL21 DE3 cells (Stratagene) after induction with 1 mM isopropyl-β-D-thiogalactopyranoside (IPTG) and purified as described for Agl3 (Meyer et al. 2011).

### Agl3 in vitro assay and saturation kinetics measurements

The conversion of UDP-d-glucose to UDP-d-glucose-5,6-*ene* catalyzed by Agl3 was performed in 20 mM 1,3-bis(tris-(hydroxymethyl)methylamino)propane (Bis–Tris propane) buffer, pH 6.5, containing UDP-d-glucose at a final concentration of 0.1, 0.2, 0.5, 1.0, 2.0, 4.0, and 10 mM, respectively, and 20 µg of purified Agl3 in a total volume of 40 µl of reaction mixture. The reaction was carried out for 30 min at 70 °C. Subsequently, the reaction mixture was rapidly cooled on ice and both the substrate and reaction product were extracted by the addition of 40 µl of chloroform followed by vortexing the mixture for 1 min. Phase separation was performed using a table centrifuge (Eppendorf 5804R) and the water phase was analyzed by RP-HPLC (Thermo Scientific/Dionex; Ultimate 3000 Standard LC System) on a Nucleosil 120–3 C18 column (Macherey–Nagel) with a flow rate of 0.6 ml min^−1^ and 0.4 M phosphate (pH 6.1) (Meyer et al. 2011). The formation of UDP-d-glucose-5,6-*ene* was investigated by PGC-ESI–MS(MS) analysis after pre-purification of the intermediate on a porous graphitized carbon (PGC) cartridge (Thermo Scientific) (Pabst et al. 2010). The conversion of UDP-d-glucose and sulfite to UDP-sulfoquinovose by Agl3 was measured under the same conditions by varying UDP-d-glucose concentrations of 0.1, 0.3, 1, 3, 10 and 30 mM against sodium sulfite concentrations of 1.0, 3.0, 10, 30, and 100 mM.

### PGC purification and desalting of UDP-activated sugars

Prior to analysis of the in vitro reaction products of Agl3 by NMR and ESI–MS(MS), the UDP-bound intermediates from the reaction mixture were made protein-free and desalted using PGC spin-prep columns (Thermo Scientific). The columns were pre-activated with 500 µl of 100 % acetonitrile and washed with 500 µl of MilliQ water prior to sample application. Bound UDP-sugars were eluted with 100 % acetonitrile and lyophilized on a SpeedVac vacuum concentrator (Pabst et al. 2010). For ESI–MS(MS) analysis, the UDP-sugars were re-dissolved in 0.3 M ammonium formate, pH 9, containing 50 % acetonitrile. The samples were measured via direct infusion on a Bruker maXis 4G mass spectrometer in the negative ion MS scan mode. Specific values were set to: spectra rate 1.0 Hz, low mass 300 *m*/*z*, ion transfer time 85 µs, pre pulse storage 10.0 µs.

### NMR analysis of the Agl3 product

Spectra were recorded at 297 K in 99.9 % D_2_O (0.6 ml) on an Avance III 600 spectrometer (Bruker; ^1^H at 600.13 MHz, ^13^C at 150.9 MHz, ^31^P at 242.9 MHz), using standard Bruker NMR software. ^1^H NMR spectra were referenced to 2,2-dimethyl-2-silapentane-5-sulfonic acid (*δ* 0.0), ^13^C NMR spectra were referenced to external dioxane (*δ* 67.40), and ^31^P spectra were referenced to external ortho-phosphoric acid (*δ* 0.0) for solutions in D_2_O. Gradient-selected ^1^H, ^1^H total correlated spectroscopy (TOCSY, mixing time 120 ms) and COSY experiments were recorded using the programs mlevph and cosygpqf, respectively, with 2048 × 256 data points and 16 and 8 scans, respectively per t1-increment. The multiplicity edited heteronuclear single quantum coherence spectra (HSQC) (Schleucher et al. 1994) were measured using the program hsqcedetgp with 1024 × 128 data points and 128 scans per t1-increment. Heteronuclear multiple bond correlation spectra (HMBC) (Bax and Summers 1986) were acquired using the pulse program hmbcgpndqf with 4096 × 64 data points and 1600 scans per t1-increment and spectral widths of 7.7 ppm for ^1^H and 222 ppm for ^13^C to check for any carbonyl correlated signals.

### Labeling with deuterium oxide

The conversion of UDP-d-glucose to UDP-d-glucose-5,6-*ene* was carried out in 20 mM phosphate buffer, prepared in D_2_O, at a pD value of 6.4 (Gabriel and Lindquist 1968). Briefly, an acidic and a basic phosphate stock solution in D_2_O were prepared by dissolving 0.037 g of Na_2_HPO_4_ and 0.035 g of NaH_2_PO_4_ in 10 ml of D_2_O, each, at a final concentration of 20 mM, and the pD values of D_2_O-prepared phosphate solutions were determined using a pH-meter (Mettler MP-220); approximate values were 5.3 and 8.8 for the acidic and the basic phosphate stock solution, respectively. The acidic and basic phosphate stock solutions were mixed at a ratio of 3:1 (v/v) to obtain a 20 mM phosphate buffer in D_2_O in the range of pD 6.4. For maximum removal of protons from the phosphate solutions, the solvents were dried using the SpeedVac concentrator and either phosphate salt was re-dissolved in 1 ml of D_2_O. Agl3 was prepared in D_2_O as follows: 2 ml of Agl3 solution (1 mg protein), purified by nickel affinity chromatography and dialyzed against 10 mM phosphate buffer, pH 6.5, were concentrated to 100 µl using the SpeedVac concentrator and supplemented with D_2_O to a final volume of 1 ml, corresponding to a protein concentration of 1 mg ml^−1^.

### Cofactor extraction and spectrophotometric analysis

Purified Agl3 (5.6 mg of protein in 10 ml of 5 mM phosphate buffer, pH 6.5) was added to 5 ml of chloroform and mixed for 1 h at room temperature (22 °C). Phase separation was done using a table centrifuge and the water phase was transferred to a new tube. Washing of the chloroform phase was repeated with 2 ml of MilliQ water. The combined water phases were concentrated to 1 ml (SpeedVac) and analyzed for the presence of the NAD-cofactor by RP-HPLC (as described above) (Meyer et al. 2011). The absorption spectrum of fractions containing NAD^+^ was measured on a diode array photometer (Agilent) in 100-µl quartz cuvettes. The in vitro reduction of extracted NAD^+^ was performed qualitatively by supplementing trace amounts of glucose dehydrogenase (Amano) and glucose (1 mM final concentration) to the NAD-cofactor and incubating the mixture at room temperature for 2 and 5 min, respectively.

### Conventional stopped-flow spectroscopy

Transient-state measurements were made using the SX.18 MV stopped-flow spectrophotometer (Applied Photophysics, Leatherhead, Surrey, UK), equipped with a 1 cm observation cell. In a typical experiment, UDP-sulfoquinovose synthase was mixed with UDP-d-glucose at 65 °C and first data points recorded starting with 1.5 ms. Final concentrations were 2 µM of Ag13 and 0.5, 5, 25 and 50 µM of UDP-d-glucose, respectively. The reduction of NAD^+^ was followed at 320 nm. Calculation of pseudo-first-order rate constants (*k*_obs_) from experimental time traces was performed with a SpectraKinetic work station (Version 4.38) interfaced to the instruments. The second-order rate constant was calculated from the slope of the linear plot of the pseudo-first-order rate constants versus substrate concentration. To follow spectral transitions, a Model PD.1 photodiode array accessory (Applied Photophysics) connected to the stopped-flow machine together with XScan diode array scanning software (Version 1.07) was utilized.

### Circular dichroism (CD) measurements

Electronic circular dichroism spectroscopy was performed using Chirascan (Applied Photophysics, Leatherhead, UK). First, the instrument was flushed with nitrogen at a flow rate of 5 l min^−1^. Then, ECD spectra were recorded at room temperature in the far-UV region (i.e., 180–260 nm). The path length was 1 mm, spectral band width 3 nm and scan time per point 10 s.

## Electronic supplementary material

Below is the link to the electronic supplementary material.

Supplementary material 1 (JPEG 2122 kb)

Supplementary material 2 (DOCX 30 kb)

## Abbreviations

Agl3, SQD1: UDP-sulfoquinovose synthases
CD: Circular dichroism spectrometry
COSY: Correlated spectroscopy
D_2_O: Deuterium oxide
ESI MS: Electrospray ionization mass spectrometry
GalE: UDP-glucose/galactose 4-epimerase
HMBC NMR: Heteronuclear multiple bond correlation nuclear magnetic resonance spectroscopy
HSQC: Heteronuclear single quantum coherence
MilliQ: ‘Ultrapure’ water (typically 18.2 MΩ·cm resistivity at 25 °C)
NAD^+^/NADH: Oxidized/reduced nicotinamide adenine dinucleotide
PGC: Porous graphitized carbon
Quip6S: Sulfoquinovose (6-deoxy-6-*C*-sulfo-d-glucopyranose)
RmlB: dTDP-d-glucose 4,6-dehydratase
SDR: Short-chain dehydrogenase/reductase enzyme superfamily
SlaA: Major surface (S-)layer protein of *Sulfolobus acidocaldarius*
TOCSY: Total correlated NMR spectroscopy
UDP-d-glucose: Uridine 5´-diphospho-d-glucose
UDP-d-glucose-5,6-*ene*: UDP 6-deoxy-d-*xylo*-hex-5-enose
