# Supplementary figures and images for: UDP-sulfoquinovose formation by Sulfolobus acidocaldarius
Source: Extremophiles. 2015 Jan 21;19(2):451–67. doi: 10.1007/s00792-015-0730-9 (PMC4388408; doi:10.1007/s00792-015-0730-9)

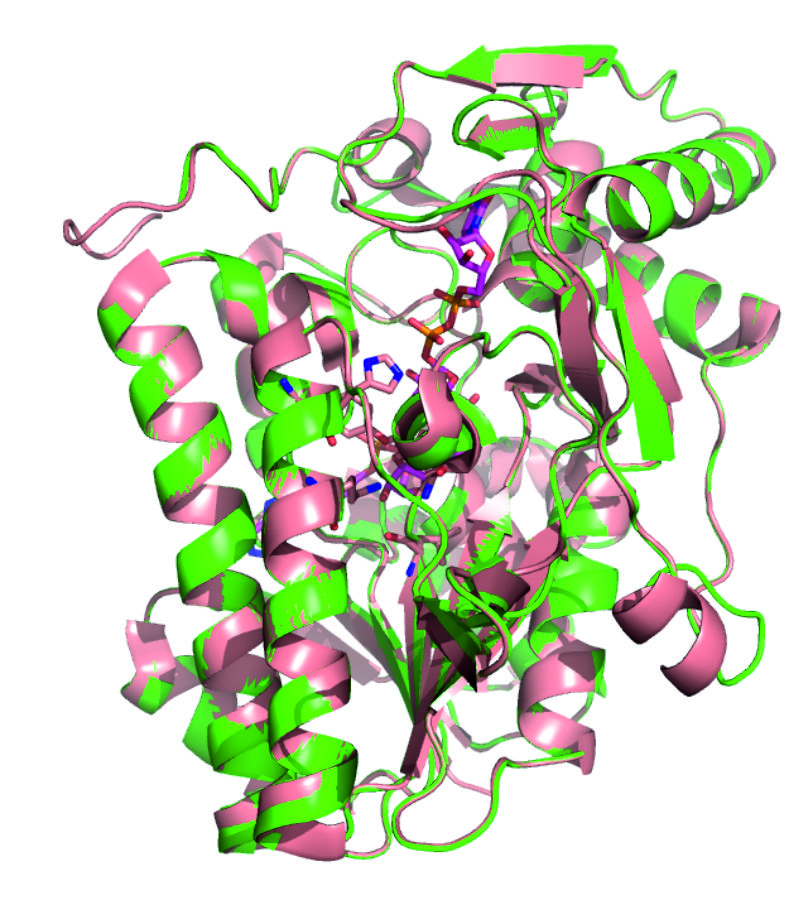

Supplement: Supplementary file 1 — Supplementary material 1 (JPEG 2122 kb) [file 792_2015_730_MOESM1_ESM.jpg]
