# Supplementary material for: UDP-sulfoquinovose formation by Sulfolobus acidocaldarius
Source: Extremophiles. 2015 Jan 21;19(2):451–67. doi: 10.1007/s00792-015-0730-9 (PMC4388408; doi:10.1007/s00792-015-0730-9)
Supplement: Supplementary file 2 — Supplementary material 2 (DOCX 30 kb) [file 792_2015_730_MOESM2_ESM.docx]

**Supplemental information**

**Procedures**

Modeling of the structure of Agl3 and its active site

A homology model of Agl3 was constructed using Swiss-Model in the automated mode (Guex and Peitsch 1997; Schwede et al. 2003; Arnold et al. 2006). As a template, the 1.6-Å resolution crystal structure of the SQD1 enzyme with Protein DataBank (Berman et al. 2000) identification code 1QRR was used (Mulichak et al. 1999). Coordinates for the NAD^+^ cofactor and the substrate uridine 5’-diphospho-glucose were transferred from the template structure after a backbone superposition of the template and the model.

**Results and Discussion**

Model structure of Agl3

Since no crystal structure of Agl3 is available, we chose an approach for the prediction of functional epitopes similar to that used for SQD1 from *A. thaliana* (Essigmann et al. 1999) before a high resolution crystal structure of that protein was available (Mulichak et al. 1999). The 1.6-Å resolution structure of the UDP-sulfoquinovose synthase SQD1, co-crystallized with NAD^+^ and UDP-D-glucose (Mulichak et al. 1999), served as a template for a homology model of the Agl3 enzyme (supplemental Figure S1). Agl3 showed 43% sequence identity with SQD1. The backbone root-mean-square deviation of the model with respect to the template structure amounts to 0.57 Å, indicating a very similar overall fold. This concurs with results when the unpublished 1.2-Å resolution structure with PDB identification code 1I24 was used as a template (data not shown).

**References**

Arnold K, Bordoli L, Kopp J, Schwede T (2006) The SWISS-MODEL workspace: a web-based environment for protein structure homology modelling. Bioinformatics 22:195-201

Berman HM, Westbrook J, Feng Z, Gilliland G, Bhat TN, Weissig H, Shindyalov IN, Bourne PE (2000) The protein data bank. Nucleic Acids Res 28:235-242

Essigmann B, Hespenheide BM, Kuhn LA, Benning C (1999) Prediction of the active-site structure and NAD^+^ binding in SQD1, a protein essential for sulfolipid biosynthesis in *Arabidopsis*. Arch Biochem Biophys 369:30-41

Guex N, Peitsch MC (1997) SWISS-MODEL and the Swiss-PdbViewer: an environment for comparative protein modeling. Electrophoresis 18:2714-2723

Mulichak AM, Theisen MJ, Essigmann B, Benning C, Garavito RM (1999) Crystal structure of SQD1, an enzyme involved in the biosynthesis of the plant sulfolipid headgroup donor UDP-sulfoquinovose. Proc Natl Acad Sci USA 96:13097-13102

Schwede T, Kopp J, Guex N, Peitsch MC (2003) SWISS-MODEL: an automated protein homology-modeling server. Nucleic Acids Res 31:3381-3385
